# Supplementary material for: A conserved regulator controls asexual sporulation in the fungal pathogen Candida albicans
Source: Nat Commun. 2020 Dec 4;11:6224. doi: 10.1038/s41467-020-20010-9 (PMC7718266; doi:10.1038/s41467-020-20010-9)
Supplement: Supplementary file 1 — Supplementary Information [file 41467_2020_20010_MOESM1_ESM.pdf]

A conserved regulator controls asexual sporulation in the fungal pathogen *Candida albicans*

SUPPLEMENTARY INFORMATION

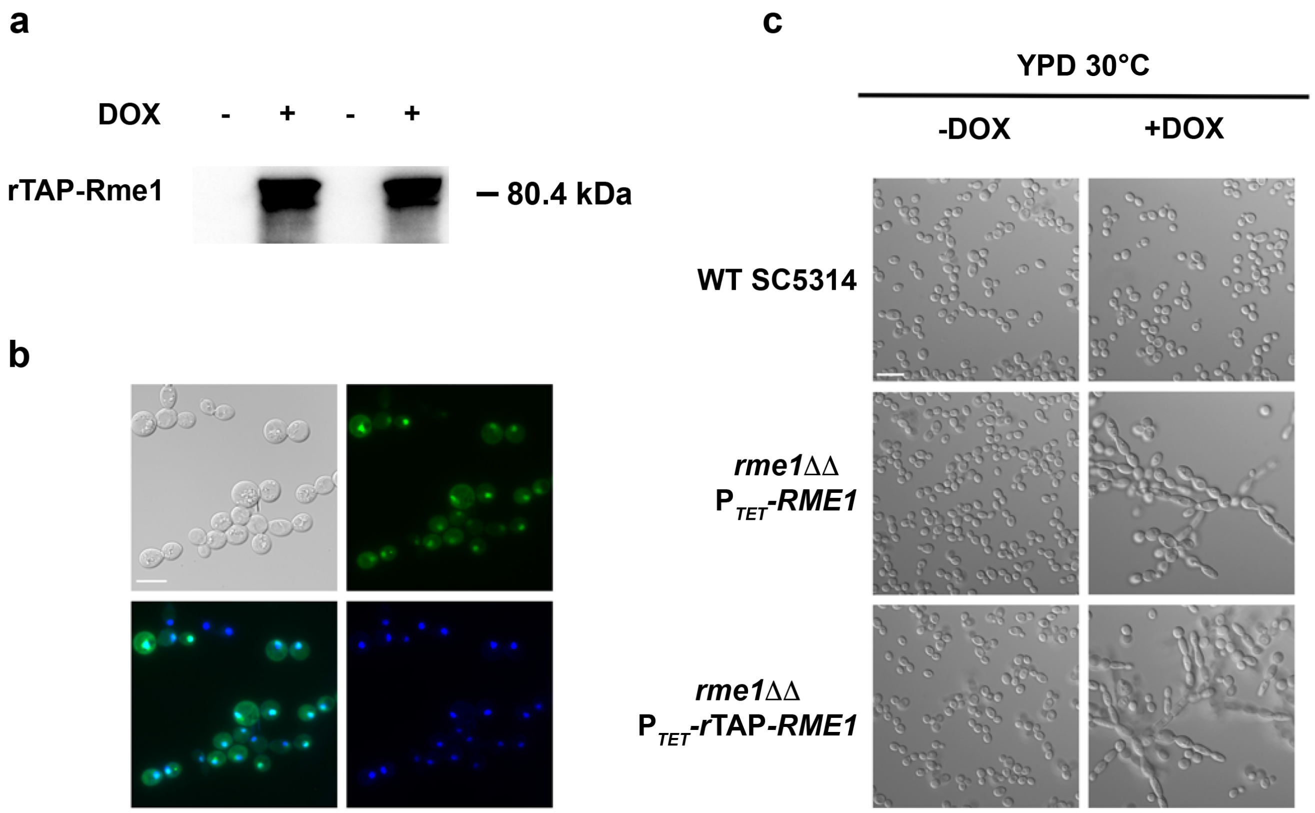

Supplementary Fig. 1. Characterization of *C. albicans* strains expressing the rTAP-tagged and untagged versions of Rme1. a) Western blot analysis of *rme1*ΔΔ mutant expressing P_{TET}-rTAP-*RME1*. Two independent clones were cultured in YPD liquid medium in the presence or absence of doxycycline, and then total protein extracts were subjected to SDS-PAGE. Western blotting was conducted using an anti-TAP polyclonal antibody to detect rTAP-Rme1. **b)** Localization of GFP-Rme1 fusion protein. A strain containing P_{TET}-*GFP-RME1* (CEC5252) was grown for 6h in YPD + 40 μg/mL doxycycline. Cells were incubated with 10 μg/mL Hoechst for 15 min and rinsed with PBS prior to imaging. Top left panel: Nomarski; Top right panel: GFP-Rme1; Bottom right panel: Hoechst staining of the nuclei; Bottom left panel: overlay. Scale bar = 10 μm. **c)** Phenotypic characterization of the strains expressing the rTAP-tagged and untagged versions of Rme1. The WT strain SC5314 (top panels) and *rme1*ΔΔ strains with an inducible Rme1 either untagged (middle panels) or fused to a N-terminal rTAP-tag (bottom panels) were grown in YPD liquid medium at 30ºC without induction (left panels) or in the presence of 40 μg/mL doxycycline (right panels). Scale bar = 10 μm.

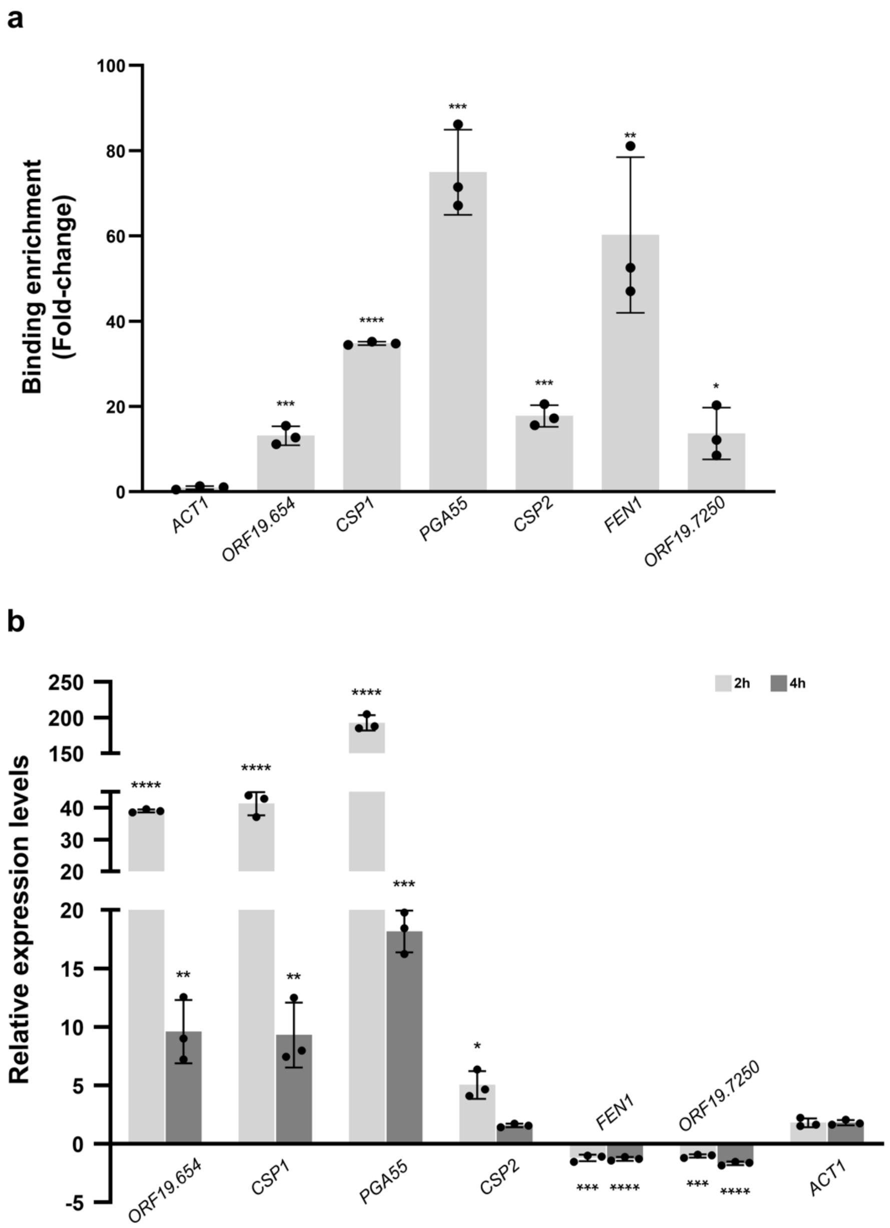

Supplementary Fig. 2. Validation of the data obtained in the ChIP-chip and transcriptomics analysis. a) Quantification of DNA enrichment after the immunoprecipitation of rTAP-tagged Rme1 at the promoters of *ORF19.654* (*P*=0.000701), *CSP1* (*P*<0.000001), *PGA55* (*P*=0.000213), *CSP2* (*P*=0.000344), *FEN1* (*P*=0.00493) and *ORF19.7250* (*P*=0.022279) by qPCR analysis in strains expressing the tagged and untagged versions of Rme1. Bars represent relative enrichment values (n-fold) of rTAP-Rme1 coimmunoprecipitated DNA as compared to DNA from mock immunoprecipitation; the *ACT1* locus was used as a reference. Data are expressed as the mean ± SD, n=3 biological replicates, over 3 experiments. **b)** The relative expression levels of the *ORF19.654* (*P*<0.000001; *P*=0.007677), *CSP1* (*P*=0.000048; 0.009535), *PGA55* (*P*=0.000006; 0,000097), *CSP2* (*P*=0.010644; 0.173319), *FEN1* (*P*=0.000368; 0.000039), *ORF19.7250* (*P*=0.000257; 0.000022) and *ACT1* (negative control) genes were quantified by RT-qPCR by using total RNA from independent clones expressing P_{TET}-*RME1* at 2h (light grey bars) and 4h (dark grey bars). The bars represent the average relative change in RNA abundance of the indicated genes in doxycycline-treated samples as compared to those that were untreated. Data are expressed as the mean ± SD, n=3 biological replicates, over 3 experiments. (*) *P*<0.05, (**) *P*<0.01, (***) *P*<0.001, (****) *P*<0.0001.

```
Ca     1 MFSYNLESNNAGYLNHHHSRHHLNSNSNSNNNNNNNNNNSIAISNNNKAHLQEQKQRQSQ
Cd     1 MFSYNLESNNAGYLNNHHSLHH-HHHHNNNNNNNNNNTSSITTNNKSKAHLQEQKQRQNQ
Cb     1 MLAYTS--------------------------DSKEQQQDQQQQQQQQQQQQQQQQQLQQ
Ct     1 MVGYNL----------------------------------IESNSINLAHIQD-----NE
Cp     1 MSPLTAIL---------------QSYHAKVEDESSIYHLLKKSGSQPSLSIDEVVVRVPS

Ca    61 EHEQNPRNPQVYQNYHFIQQQQHFQYLQ----------NALANTMSQLQHH---------
Cd    60 EHEQNPRNPQVYQNYHFIQQQQHFQYLQ----------NALANTMSQLQHH---------
Cb    35 QQQQQQQQQQQQYNYHFIQQQQHFQYLQ----------NALANTMNQINH----------
Ct    22 QHQEPNSHSHLYHHYQFVQQQQHFQYLQ----------NALASTMSQQQQQQLPDQQDSQ
Cp    46 VGEPSFTCQSAMLLNELLAKASNHQSIEGIGTTQIRPVSALFGAKDRIADNSTIELVNHH

Ca   102 --------PPYH-GHAVFKPNYMQDVFLNDSCSLGSPVNSIENSGCTTTKTTPIISPM--
Cd   101 --------PPYH-GHAVFKPNYMQDVFLNDSCSLGSPVNSIENSGCTTTKTTPIIPPM--
Cb    75 --------PPYF-MNSVFKPNYMQDVLLNDSCSLDSPSVNYTDSNQTTTKTTPIITPI--
Ct    72 DQPNSQSPSPYHPSSAVFKPNYMQDVLLNETCILNSPMNSMDTSACTTTKTTPLMHPI--
Cp   106 YSQLSTSYPDKSTGFMVYGKCEIDNTLLHDPETLRNPSSSSSMSSANTVLSSSPVSQMYQ

Ca   151 ------------------SLN---D-NVLPPPNHHDFDTF-MGNNFV------DYTSSYN
Cd   150 ------------------SLN---D-NVLPPPNHHDFETF-MGNNFV------DYTSTYN
Cb   124 ------------------SLTESIDQQHRYHNNQHEFDNF-LNHHHH---HHHHHHPQTI
Ct   130 ------------------SVH---EFDQLPLNNNHHLHHYHFPHEHANIAARSDSITTLN
Cp   166 NPDKAIEQSRLSQIQTNSELYSLVEQDLRFTPALYRMSPFAISHEVD------DHVEES-

Ca   182 PEHT--LPSPSSPLTMVCNDG--RRHDDHMNFASTFSVH------PASSKCAIQHYGNSN
Cd   181 PEHA--LPSPSSPLTMVGSDS--RRHEDYMNFASTFSVH------PASSKCAIQPNANSN
Cb   162 KDHS--LPSPLSPVTEFYNQNITKHHDDYLKYVSSFPLHTPGNFATTTTTTTTNHNNNNN
Ct   169 EDKKCILPSPSSPMTVTDGK---QPHDEYLNFVTTFSIH------PQSTSSLVFRNHHHI
Cp   219 ------FPHSICPW---------QLQSNNIIYSQPTPVH------LKLKRGLLENIQDVF

Ca   232 QELTKGYPNPNSLVYN---TTNNCI----------NNNSNSNNNKYSSHEYKDTRMMEMA
Cd   231 QEFTKGYPNPNSLVYN---TTNNCVINRNSNTTDANNNNNNSSNKYPLIEYKDNRMMEMT
Cb   220 NILLPHKNEINPLSYN-------------------NNLTDYNNNN------KEKKLFEMN
Ct   220 NSAVRH------ASYDLGCSTSENV---------------------------------IA
Cp   258 NLTIDDDDDKGVSEYHEGSTIQDCIMCM--SVSPSISPRQYALSRLCSPKMNNLAVFKFA

Ca   279 TPESPH--KRNSLPN--TRVPIIPTSPTFPIALNLIHSNANTKRHKSLGSATSMLQPPLE
Cd   288 TPESPH--KRNSLPN--TRVPIIPTSPTFPIALNLIHSNSSAKRHKSLGSAASILQPPLE
Cb   255 TPQSSP--KKDLFAN--TKIPIIPISPTFPAALNIPHGKVSPRKMPTTNNSFTILKPSFD
Ct   241 TPESSPNIRKGLFEDNSSRFPIIPSSPTFPAALNLSHGKVVPQKMPKQSGP--VLQPAFE
Cp   316 TTPAPP----ESFET-----PVAPETTPVSTADISVHGTPAVDPVNNNHSDD---DDGDD

Ca   335 DSRLMN-----------SG------------------HAEPMKKRLLSRFSSRLIQKLRL
Cd   344 DNKLIN-----------NG------------------HAEPMKRKLLSRFSSRLIQKLRL
Cb   311 DNIIIKSRTNNRKNHSFGGSMKQI---NL--------FAGSMKQKLLDKFSVRLLERIKL
Ct   299 IKNMTTIKKINPEQSAATGRSKSYSRKNVNTANVLSQGYESMKKRLLARYSTRLLQRMRL
Cp   364 DDDITPHPTEATSKASWTNSSDFC---NAATSGIDASKLEEYKKAFFSQYSKVSITKIKV

Ca   366 AESDMKCRKHCNTRFGNYLELIDHFEGHGLQRYLSSRTFICPVKECPMNMIGFDKRAELR
Cd   375 AESDMKCRKHCNTRFGNYLELIDHFEGHGLQRYLSSRTFICPVKECPMNMIGFDKRAELR
Cb   360 AENDIKCRKHCDIKFANCLELIDHYESYGLHENLQSRSFLCPVKECPLNILGFDKKADLR
Ct   359 SESDTKCRKHCEVDFTNYLELINHYEDNGLQMHLETRNFKCPVKECPMNIIGFDKRADLR
Cp   421 AESDKKFLPKNDSK-PNYYTLVDNLDIFTCSKLACTKRYKCPVKECPMHFLGIKKRAELK

Ca   426 HHVHSDHVTHGLVSIQYAKYSEEIKEFLFVCDEENCGKGFYRSDTLTRHIKLVHKREKHF
Cd   435 HHVHSDHVTHGLVSIQYAKYSKEIKEFLFVCDEENCGKGFYRSDTLTRHIKLVHKREKHL
Cb   420 HHVHSEHLTHGLVSVQYAKYANELEKFLFVCDEPNCGKGFYRSDTLTRHVKLVHKRDKQP
Ct   419 HHVHSDHLTHGLVSAQYSKYSDEIKKYLFVCDEPSCGKGFYRSDTLTRHVKLVHKRTSNF
Cp   480 HHVHYEHLKNGFVKLGCREYEDEIMRILFVCNEAGCGKAFYRCDSLNRHLHLVHGNKRKG

Ca   486 TKRKRRQVVAHQEDKA----IKKSKG-------------------------
Cd   495 TKRKRRQVVAHQDDKA----IKKSRS-------------------------
Cb   480 IRRKRKFSNNNNNNNNDKSLSKKTKN-------------------------
Ct   479 TRRRRRRINKNHA--------------------------------------
Cp   540 GGAKRKFVVNDEVELADVELVENYNIDRELNCQDDGDIEVCTTLKRRKKVI
```

Supplementary Fig. 3. Multiple alignment of Rme1 sequences. MUSCLE69 was used to generate a multiple alignment of the Rme1 protein sequences from *C. tropicalis* (CTRG_03993) [obtained from the NCBI (https://www.ncbi.nlm.nih.gov)], *C. parapsilosis* (Cpar2_212670p) [obtained from CGD (http://www.candidagenome.org)], *C. buenavistaensis* [*CbRME1* was PCR amplified and sequenced; the nucleotide sequence has been deposited at GenBank under the accession number MK070497], *C. dubliniensis* (Cd36_06830p), and *C. albicans* (C1_07330wp), with default parameters, without trimming. Boxshade (https://embnet.vital-it.ch/software/BOX_form.html) was used to highlight identical- and similar-residues (black and grey boxes, respectively).

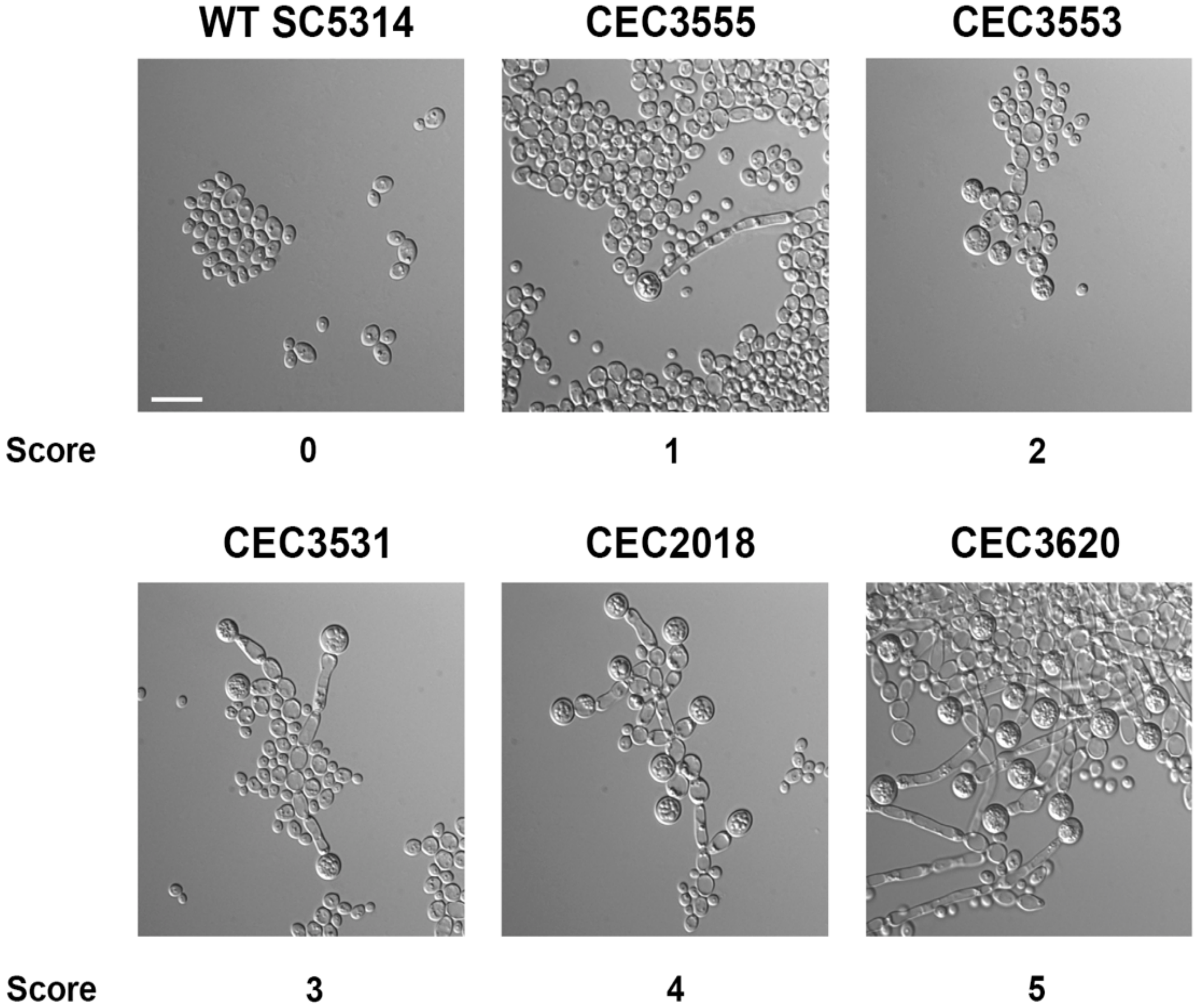

Supplementary Fig. 4. Chlamydospore formation scoring in the collection of *C. albicans* clinical isolates. Representative examples of *C. albicans* strains showing the chlamydospore formation status observed in the screen. The WT SC5314 strain is scored as 0, whereas the increase in chlamydospore formation is scored from 1 to 5. The screen was conducted in triplicate for three biological replicates. Scale bar = 10 μm.

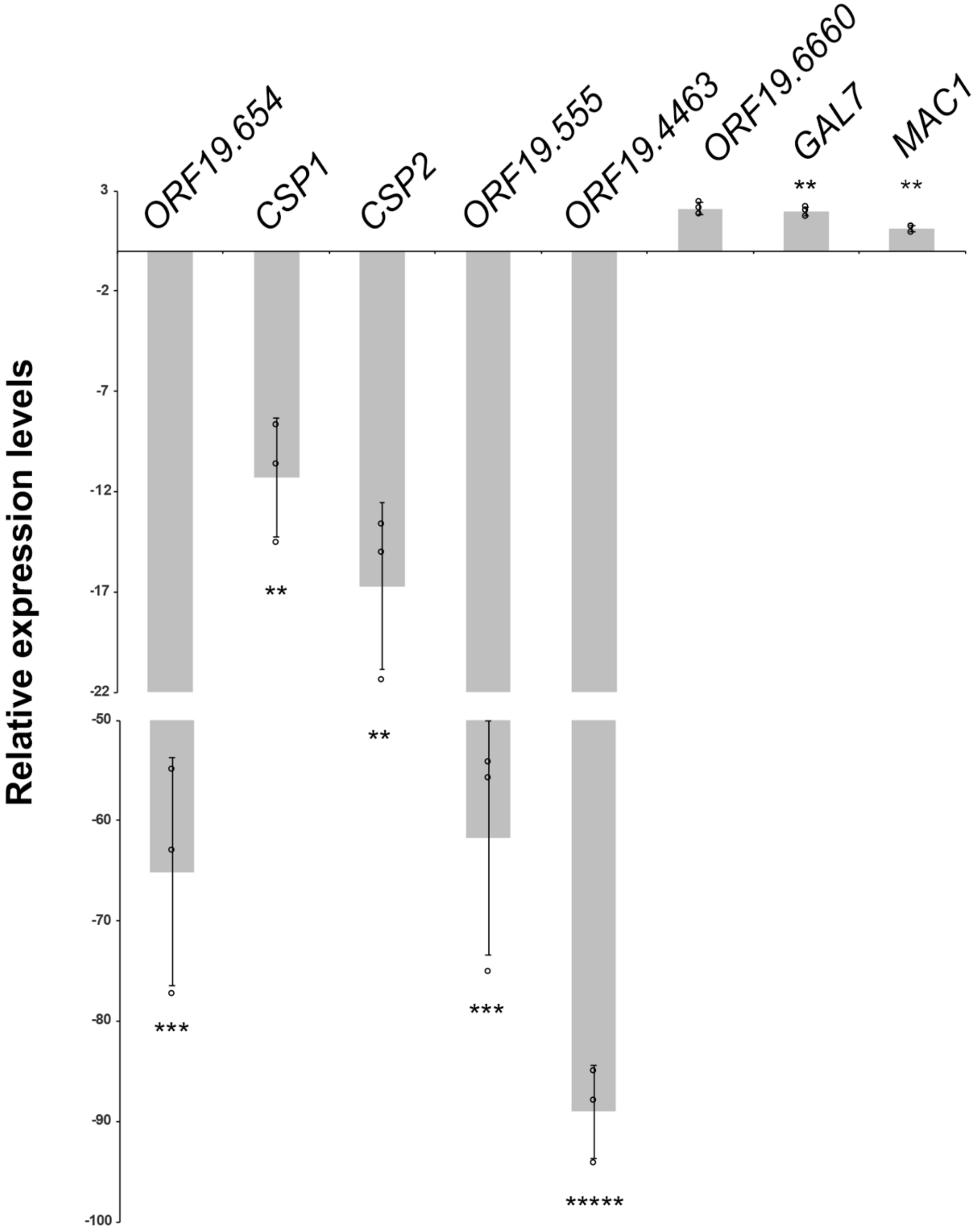

Supplementary Fig. 5. Validation of the data obtained in the microarray experiment performed in the CEC2018-*rme1*ΔΔ strain. The relative expression levels of the *IFL* family genes (*CSP1*, *CSP2*, *ORF19.654*, *ORF19.555* and *ORF19.4463*), and the *ORF19.6660* and *GAL7* genes used as negative controls were quantified by RT-qPCR using total RNA from independent CEC2018-*rme1*ΔΔ clones. *MAC1* was used as a reference. The bars represent the average change in RNA abundance of the indicated genes in the CEC2018-*rme1*ΔΔ strain as compared to those from the CEC2018 strain. *ORF19.654* (*P*=0.000537), *CSP1* (*P*=0.001957), *CSP2* (*P*=0.00173), *PGA55* (*P*=0.000732), *ORF19.4463* (*P*=0.0000047), *ORF19.6660* (*P*=0.00624) and *GAL7* (*P*=0.00677). Data are expressed as the mean ± SD, n=3 biological replicates, over 3 experiments. (*) $P<0.05$, (**) $P<0.01$, (***) $P<0.001$, (****) $P<0.0001$.

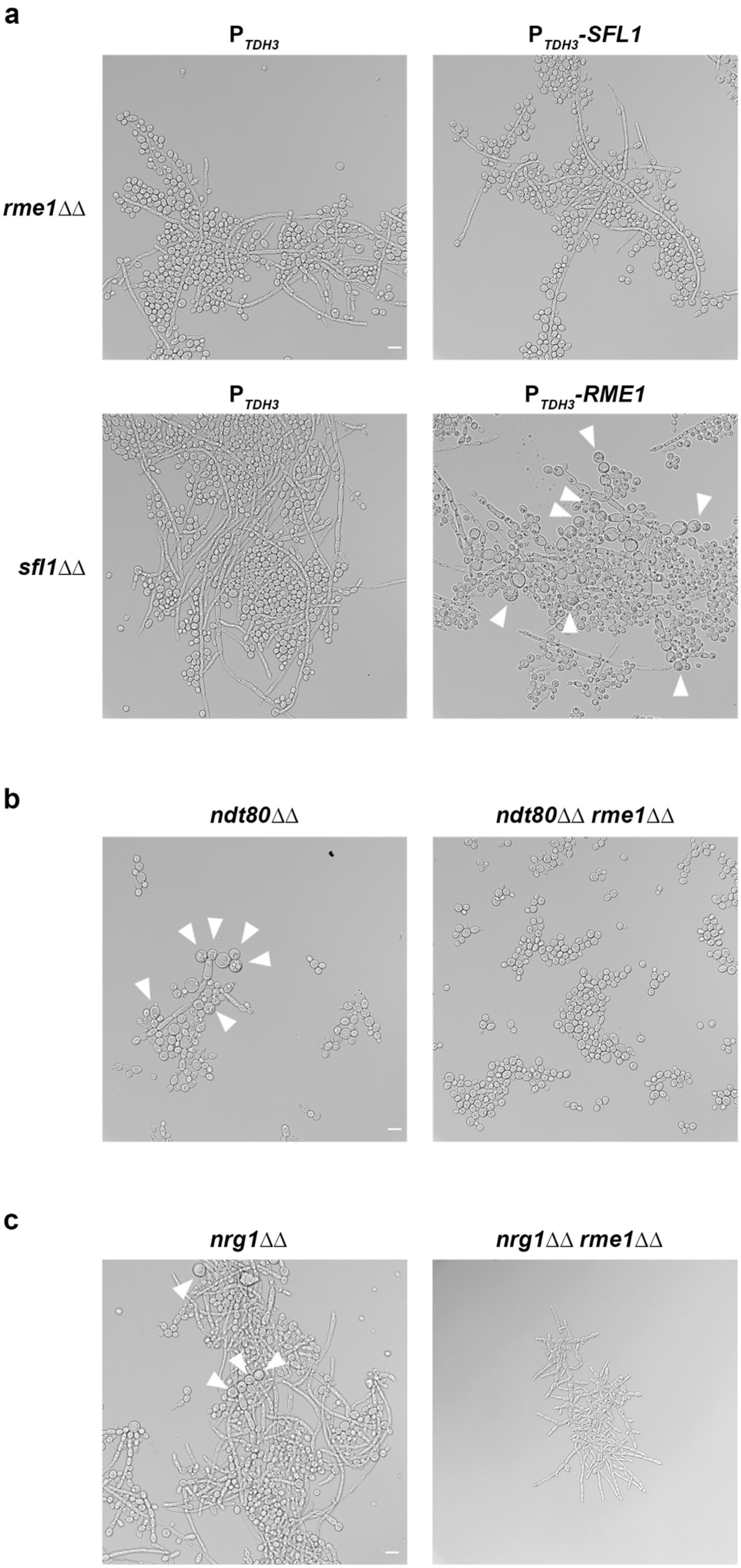
a
P_{TDH3}
P_{TDH3}-*SFL1*
*rme1*ΔΔ
P_{TDH3}
P_{TDH3}-*RME1*
*sfl1*ΔΔ
b
*ndt80*ΔΔ
*ndt80*ΔΔ *rme1*ΔΔ
c
*nrg1*ΔΔ
*nrg1*ΔΔ *rme1*ΔΔ

Supplementary Fig. 6. *RME1* acts downstream of *SFL1, NDT80* and *NRG1*. a) Strains constitutively overexpressing either *SFL1* (top right panel, P_{TDH3}-*SFL1*) or *RME1* (bottom right panel, P_{TDH3}-*RME1*) in the *rme1*Δ/*rme1*Δ (*rme1*ΔΔ) or the *sfl1*Δ/*sfl1*Δ (*sfl1*ΔΔ) strain backgrounds, respectively, were cultured overnight in liquid chlamydospore-inducing conditions together with the parental control strains *rme1*Δ/*rme1*Δ and *sfl1*Δ/*sfl1*Δ transformed with the empty overexpression plasmid (top and bottom left panels, P_{TDH3}) before being examined by light microscopy for chlamydospore formation (white arrowheads). Scale bar=10µm. **b)** The *C. albicans ndt80*Δ/*ndt80*Δ (*ndt80*ΔΔ, left panel) together with the *ndt80*Δ/*ndt80*Δ *rme1*Δ/*rme1*Δ double mutant (*ndt80*ΔΔ *rme1*ΔΔ, right panel) strains as well as **c)** the *C. albicans nrg1*Δ/*nrg1*Δ (*nrg1*ΔΔ, left panel) and *nrg1*Δ/*nrg1*Δ *rme1*Δ/*rme1*Δ double mutant (*nrg1*ΔΔ *rme1*ΔΔ, right panel) were similarly grown in chlamydospore-inducing conditions before being microscopically examined for their efficiency to form chlamydospores (white arrowheads). Scale bar=10µm.

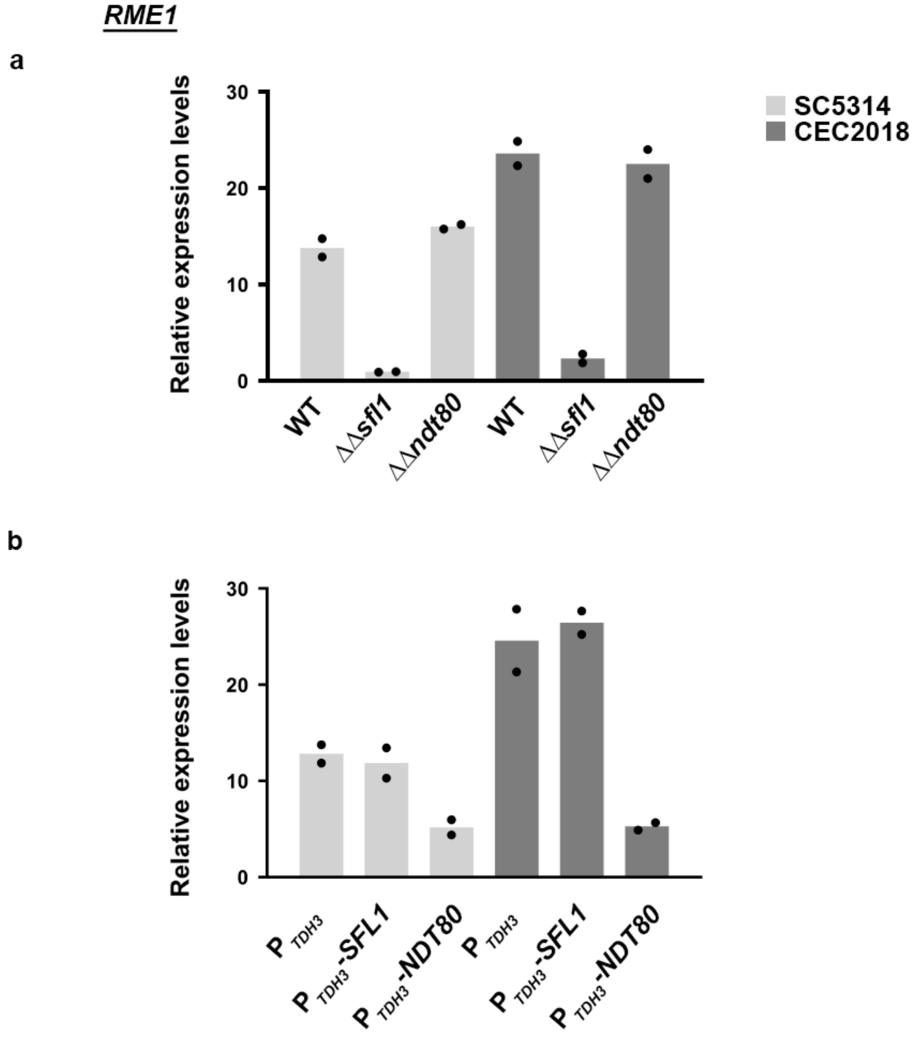

Supplementary Fig. 7. Sfl1 and Ndt80 antagonistically affect expression of *RME1*. a) Mutant strains *sfl1*ΔΔ and *ndt80*ΔΔ in SC5314 or CEC2018 backgrounds, and **b)** strains constitutively overexpressing *SFL1* or *NDT80* in SC5314 or CEC2018 backgrounds were grown overnight at 25ºC in liquid chlamydospore-inducing conditions. Total RNA was extracted and the relative expression levels of *RME1* were determined by RT-qPCR using *ACT1* as a calibrator. n=2 biological replicates, over 3 experiments.

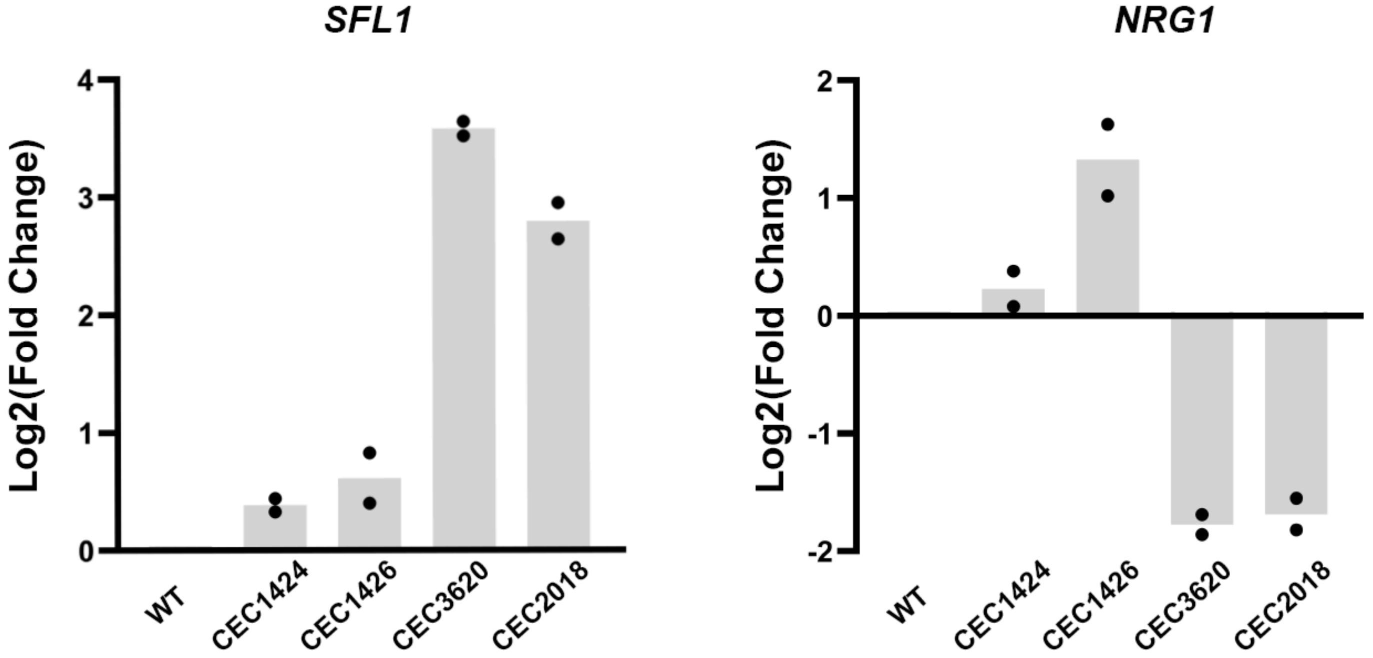

Supplementary Fig. 8. *SFL1* and *NRG1* expression levels correlate with the efficiency of clinical isolates to form chlamydospores. Relative expression levels of *SFL1* and *NRG1* were determined by RT-qPCR in *C. albicans* clinical isolates and the reference strain SC5314 (WT control). CEC1424 and CEC1426 are defective for chlamydospore formation, whereas CEC3620 and CEC2018 are efficient for chlamydospore formation (*x*-axes). Log_2-transformed expression levels (fold change, *y*-axes) of *SFL1* (left) and *NRG1* (right) in the indicated strains relative to their expression in the reference strain SC5314 are shown on the *y*-axes. n=2 biological replicates, over 3 experiments.

SUPPLEMENTARY REFERENCES

1. Gillum, A. M., Tsay, E. Y. & Kirsch, D. R. Isolation of the *Candida albicans* gene for orotidine-5′-phosphate decarboxylase by complementation of *S. cerevisiae ura3* and *E. coli pyrF* mutations. *Mol. Gen. Genetics* **198,** 179–182 (1984).

2. Wilson, R., Davis, D. & Mitchell, A. Rapid hypothesis testing with *Candida albicans* through gene disruption with short homology regions. *J. Bacteriol.* **181,** 1868–74 (1999).

3. Chauvel, M. *et al.* A Versatile Overexpression Strategy in the Pathogenic Yeast *Candida albicans*: Identification of Regulators of Morphogenesis and Fitness. *PLoS One* **7,** e45912 (2012).

4. Cabral, V. *et al.* Targeted Changes of the Cell Wall Proteome Influence *Candida albicans* Ability to Form Single- and Multi-strain Biofilms. *PLoS Pathog.* **10,** e1004542 (2014).

5. Noble, S. M. & Johnson, A. D. Strains and Strategies for Large-Scale Gene Deletion Studies of the Diploid Human Fungal Pathogen *Candida albicans*. *Eukaryot. Cell* **4,** 298–309 (2005).

6. Odds, F. C. *et al.* One year prospective survey of *Candida* bloodstream infections in Scotland. *J. Med. Microbiol.* **56,** 1066–1075 (2007).

7. Bougnoux, M.-E. *et al.* Candidemia and candiduria in critically ill patients admitted to intensive care units in France: incidence, molecular diversity, management and outcome. *Intens. Care Med.* **34,** 292–299 (2008).

8. Ropars, J. *et al.* Gene flow contributes to diversification of the major fungal pathogen *Candida albicans*. *Nat. Commun.* **9,** 2253 (2018).

9. Enjalbert, B. *et al.* Role of the Hog1 Stress-activated Protein Kinase in the Global Transcriptional Response to Stress in the Fungal Pathogen *Candida albicans*. *Mol. Biol. Cell* **17,** 1018–1032 (2006).

10. Lo, H.-J. *et al.* Nonfilamentous *C. albicans* Mutants Are Avirulent. *Cell* **90,** 939–949 (1997).

11. Znaidi, S., Nesseir, A., Chauvel, M., Rossignol, T. & d'Enfert, C. A Comprehensive Functional Portrait of Two Heat Shock Factor-Type Transcriptional Regulators Involved in *Candida albicans* Morphogenesis and Virulence. *PLoS Pathog.* **9,** e1003519 (2013).

12. Morschhäuser, J., Ruhnke, M., Michel, S. & Hacker, J. Identification of CARE-2-negative *Candida albicans* isolates as *Candida dubliniensis*. *Mycoses* **42,** 29–32 (1999).

13. Suh, S., Nguyen, N. H. & Blackwell, M. Yeasts isolated from plant-associated beetles and other insects: seven novel Candida species near *Candida albicans*. *FEMS Yeast Res.* **8,** 88–102 (2008).

14. Zeidler, U. *et al.* Synergy of the antibiotic colistin with echinocandin antifungals in *Candida* species. *J. Antimicrob. Chemoth.* **68,** 1285–1296 (2013).

15. Bougnoux, M.-E., Morand, S. & d'Enfert, C. Usefulness of Multilocus Sequence Typing for Characterization of Clinical Isolates of *Candida albicans*. *J. Clin. Microbio.l* **40,** 1290–1297 (2002).

16. Sdoudi, K. *et al.* Phylogeny and Diversity of *Candida albicans* Vaginal Isolates from Three Continents. *Int. J. Curr. Microbiol. App. Sci.* **3,** 471–480 (2014).

17. Schönherr, F. *et al.* The intraspecies diversity of *C. albicans* triggers qualitatively and temporally

distinct host responses that determine the balance between commensalism and pathogenicity. *Mucosal Immunol.* **10,** 1335–1350 (2017).

18. Angebault, C. *et al. Candida albicans* Is Not Always the Preferential Yeast Colonizing Humans: A Study in Wayampi Amerindians. *J. Infect. Dis.* **208,** 1705–1716 (2013).

19. Shin, J. *et al.* Genetic Diversity among Korean *Candida albicans* Bloodstream Isolates: Assessment by Multilocus Sequence Typing and Restriction Endonuclease Analysis of Genomic DNA by Use of BssHII. *J. Clin. Microbiol.* **49,** 2572–2577 (2011).

20. Bougnoux, M.-E. *et al.* Multilocus Sequence Typing Reveals Intrafamilial Transmission and Microevolutions of *Candida albicans* Isolates from the Human Digestive Tract. *J. Clin. Microbiol.* **44,** 1810–1820 (2006).

21. Calderón-Noreña, D. M. *et al.* A Single Nucleotide Polymorphism Uncovers a Novel Function for the Transcription Factor Ace2 during *Candida albicans* Hyphal Development. *PLoS Genet.* **11,** e1005152 (2015).

22. Bougnoux, M.-E. *et al.* Multilocus sequence typing of *Candida albicans*: strategies, data exchange and applications. *Infect. Genetics Evol.* **4,** 243–252 (2004).
