# Supplementary material for: A conserved regulator controls asexual sporulation in the fungal pathogen Candida albicans
Source: Nat Commun. 2020 Dec 4;11:6224. doi: 10.1038/s41467-020-20010-9 (PMC7718266; doi:10.1038/s41467-020-20010-9)
Supplement: Supplementary file 3 — Description of Additional Supplementary Files [file 41467_2020_20010_MOESM3_ESM.pdf]

Description of Additional Supplementary Files

File Name: Supplementary Data 1
Description: **Strains used in this study.**

File Name: Supplementary Data 2
Description: **Primers used in this study.**

File Name: Supplementary Data 3
Description: **Complete dataset of Rme1p ChIP-chip experiment - Replicate 1 (upper array).** These are the complete Rme1p binding data from the biological replicate 1. The intersect of peak intervals from Replicates 1 and 2 (Supplementary Data 3 and 4) was used to generate Supplementary Data 5. Headers: **#rank**, Rme1p binding peak rank; **chromosome**, chromosome number according to Assembly 20 of the *C. albicans* genome; **start**, start coordinate of the peak interval; **end**, end coordinate of the peak interval; **region_length**, peak length (bp); **maxFC(log2)**, maximal log_2-transformed fold-change (IP/Control) enrichment within the peak (i.e. peak summit); **position_of_maxFC**; position of the probe corresponding to peak summit; **FDR**, false discovery rate based on MaxM/P, which is the maximal TileMap-MA statistic or maximal HMM posterior probability of all the probes within the peak (see http://www.biostat.jhsph.edu/~hji/cisgenome/index_files/manual.htm#Manual_FormatTiling for more detail).

File Name: Supplementary Data 4
Description: **Complete dataset of Rme1p ChIP-chip experiment - Replicate 2 (lower array).** These are the complete Rme1p binding data from the biological replicate 2. The intersect of peak intervals from Replicates 1 and 2 (Supplementary Data 3 and 4) was used to generate Supplementary Data 5. Headers: **#rank**, Rme1p binding peak rank; **chromosome**, chromosome number according to Assembly 20 of the *C. albicans* genome; **start**, start coordinate of the peak interval; **end**, end coordinate of the peak interval; **region_length**, peak length (bp); **maxFC(log2)**, maximal log_2-transformed fold-change (IP/Control) enrichment within the peak (i.e. peak summit); **position_of_maxFC**; position of the probe corresponding to peak summit; **FDR**, false discovery rate based on MaxM/P, which is the maximal TileMap-MA statistic or maximal HMM posterior probability of all the probes within the peak (see http://www.biostat.jhsph.edu/~hji/cisgenome/index_files/manual.htm#Manual_FormatTiling for more detail).

File Name: Supplementary Data 5
Description: **Intersect of ChIP-chip replicate1 and replicate 2.** Overlapping peak intervals (intersection) from Rme1p binding data replicates 1 and 2 (generated using the Galaxy tool Intercept version 1.0.0). N/A, indicates that binding peaks are not clearly associated with ORFs. If the peak was found in the promoter region common to two adjacent ORFs in opposite orientations, the two possible predicted target genes are shown in **Target 1** and **Target 2** columns. If only one ORF is clearly associated with a given peak, the ORF is indicated in the **Target 1** column. Headers: **Chr**, chromosome number according to Assembly 20 of the *C. albicans* genome; **Start**, start coordinate of the peak interval; **End**, end coordinate of the peak interval; **Target 1**, orf19 nomenclature according to CGD; **Target 2**, orf19 nomenclature according to CGD; **Comments**, useful comments particularly for assigning *bona fide* peaks - artifactual "binding within ORF" occurs particularly when a given gene is highly transcribed.

File Name: Supplementary Data 6
Description: **Complete dataset of RME1 transcript profiling experiments.** Headers: **Probe ID**, microarray probe identifier matching each C. albicans ORF according to the orf19 nomenclature at the Candida genome database (CGD); **Gene name**, gene name according to CGD; **Description**, gene description according to CGD; **OE 2h FC**, fold-change expression value and **OE 2h ratio**, relative expression ratio in doxycycline-treated cells versus untreated controls from an average of 3 independent biological replicates at time point 2h following induction of P_{TET}-*RME1*; **OE 2h p-student**, P-value of the corresponding fold-change values using a Student's t-test (within group); **OE 4h FC**, fold-change expression value and **OE 4h ratio**, relative expression ratio in doxycycline-treated cells versus untreated controls from an average of 3 independent biological replicates at time point 4h following induction of P_{TET}-*RME1*; **OE 4h p-student**, *P*-value of the corresponding fold-change values using a Student's *t*-test (within group).

File Name: Supplementary Data 7
Description: **Scoring of chlamydospore formation efficiency of clinical isolates.** 149 clinical isolates have been tested for their ability to form chlamydospore when grown ON in the dark in liquid PCB medium. Scores have been tested in 3 independent experiments. **Strain,** strain name. In bold, CEC4297 is OE RME1constitutively. **Type,** anatomical origin of the strain; all strains are of human origin, except those labelled "starling". **Clade,** clade derived from whole sequence analysis; NC: not claded; N/A: Not applicable. **Scoring,** scores have been given from 0 (no chlamydospores observed in 3 independent experiments) to 5 (chlamydospores formed in plenty in all 3 experiments). 1 was given when

chlamydospores were formed scarcely, 2 and 3 chlamydospore were formed in rather good numbers, and 4 when the strains repeatedly gave rise to good numbers of chlamydospore per field of view. **Reference,** articles where the isolates were first described in.

File Name: Supplementary Data 8

Description: **Complete dataset of CEC2018-*rme1*ΔΔ transcript profiling experiment.** Headers: **Probe ID,** microarray probe identifier matching each *C. albicans* ORF according to the orf19 nomenclature at the *Candida albicans* genome database (CGD); **Gene name,** gene name according to CGD; **Description,** gene description according to CGD; **Fold change**, fold-change expression value and **Ratio**, relative expression ratio in CEC2018-*rme1*ΔΔ strain versus CEC2018 strain from an average of 3 independent biological replicates at 24h incubation in PCB liquid medium; **p-student,** *P*-value of the corresponding fold-change values using a Student's *t*-test (within group).
