# Supplementary material for: A conserved regulator controls asexual sporulation in the fungal pathogen Candida albicans
Source: Nat Commun. 2020 Dec 4;11:6224. doi: 10.1038/s41467-020-20010-9 (PMC7718266; doi:10.1038/s41467-020-20010-9)
Supplement: Supplementary file 12 — Reporting Summary [file 41467_2020_20010_MOESM12_ESM.pdf]

natureresearch

Corresponding author(s): Christophe d'Enfert
Sophie Bachellier-Bassi

Last updated by author(s): Oct 23, 2020

Reporting Summary

Nature Research wishes to improve the reproducibility of the work that we publish. This form provides structure for consistency and transparency in reporting. For further information on Nature Research policies, seeAuthors & Referees and theEditorial Policy Checklist .

Statistics

For all statistical analyses, confirm that the following items are present in the figure legend, table legend, main text, or Methods section.

n/a	Confirmed	
[ ]	[x]	The exact sample size (n) for each experimental group/condition, given as a discrete number and unit of measurement
[x]	[ ]	A statement on whether measurements were taken from distinct samples or whether the same sample was measured repeatedly
[ ]	[x]	The statistical test(s) used AND whether they are one- or two-sided<br>*Only common tests should be described solely by name; describe more complex techniques in the Methods section.*
[x]	[ ]	A description of all covariates tested
[x]	[ ]	A description of any assumptions or corrections, such as tests of normality and adjustment for multiple comparisons
[ ]	[x]	A full description of the statistical parameters including central tendency (e.g. means) or other basic estimates (e.g. regression coefficient) AND variation (e.g. standard deviation) or associated estimates of uncertainty (e.g. confidence intervals)
[ ]	[x]	For null hypothesis testing, the test statistic (e.g. F, t, r) with confidence intervals, effect sizes, degrees of freedom and P value noted<br>*Give P values as exact values whenever suitable.*
[x]	[ ]	For Bayesian analysis, information on the choice of priors and Markov chain Monte Carlo settings
[x]	[ ]	For hierarchical and complex designs, identification of the appropriate level for tests and full reporting of outcomes
[x]	[ ]	Estimates of effect sizes (e.g. Cohen's d, Pearson's r), indicating how they were calculated

Our web collection on statistics for biologists contains articles on many of the points above.

Software and code

Policy information about availability of computer code

Data collection	- Openlab software version 3.5.1, Improvision Inc. or Cell sens software (Olympus) to acquire images
Data analysis	- GenePix software version 7.0 (Molecular Devices Downington, PA) to analyze ChIP-chip data, and GenePix Pro 6.1.0.2 software (Molecular Devices, Downington, PA) to generate scanned images and analyze expression microarray data.<br>- CisGenome to normalize ChIP-chip data and find peaks<br>- Arraypipe 2.0 software to normalize expression microarray data, merge replicate spots from ChIP-chip GPR files and generate the corresponding median intensity data.<br>- Integrated Genomics Viewer (IGV_2.3.68) software to visualize ChIP-chip results<br>- realplex software version 2.2 (Eppendorf®) for qPCR data analysis<br>- MUSCLE v3.8.311 for multiple alignment<br>- trimAl v32 to trim the alignments<br>- ProTest v2.43 to choose the best protein substitution models<br>- PHYML v3.0.14 to reconstruct phylogenetic trees<br>- Prism 8.4.3 (GraphPad) to draw graphs<br>- Photoshop CS6 (Adobe) to create figures

For manuscripts utilizing custom algorithms or software that are central to the research but not yet described in published literature, software must be made available to editors/reviewers. We strongly encourage code deposition in a community repository (e.g. GitHub). See the Nature Research guidelines for submitting code & software for further information.

Data

Policy information about availability of data

All manuscripts must include a data availability statement. This statement should provide the following information, where applicable:

- Accession codes, unique identifiers, or web links for publicly available datasets
- A list of figures that have associated raw data
- A description of any restrictions on data availability

Sequence data that support the findings of this study have been deposited in GenBank under the access number MK070497.
The data that support the findings of this study are available at the Gene Expression Omnibus repository with the accession numbers GSE142159 and 142370.

Field-specific reporting

Please select the one below that is the best fit for your research. If you are not sure, read the appropriate sections before making your selection.

☒ Life sciences ☐ Behavioural & social sciences ☐ Ecological, evolutionary & environmental sciences

For a reference copy of the document with all sections, see nature.com/documents/nr-reporting-summary-flat.pdf

Life sciences study design

All studies must disclose on these points even when the disclosure is negative.

Sample size	No sample size calculation was performed. Our study was based on the assumption that at least 3 independent biological replicates are sufficient to calculate statistical significance. On the other hand, based on the fact that off-target effects may occur during mutant construction in C. albicans, we usually construct mutants two times independently and verify the reproducibility of phenotypes.
Data exclusions	No data were excluded from the analyses
Replication	Experiments were replicated (or performed) at least 3 times independently. All attempts at replication were successful (or yielded statistically significant differences). When building mutant strains, several transformants were assessed for phenotypes.
Randomization	No random sampling was performed since there were no selection steps with the potential for introducing bias in our study.
Blinding	Since no randomization was used, blinding experiments were not relevant in our case. For chlamydosporulation scoring (Figure S4), scores were assessed by two independent observers.

Reporting for specific materials, systems and methods

We require information from authors about some types of materials, experimental systems and methods used in many studies. Here, indicate whether each material, system or method listed is relevant to your study. If you are not sure if a list item applies to your research, read the appropriate section before selecting a response.

Materials & experimental systems

n/a	Involved in the study	
☐	☒	Antibodies
☒	☐	Eukaryotic cell lines
☒	☐	Palaeontology
☐	☒	Animals and other organisms
☒	☐	Human research participants
☒	☐	Clinical data

Methods

n/a	Involved in the study	
☒	☐	ChIP-seq
☒	☐	Flow cytometry
☒	☐	MRI-based neuroimaging

Antibodies

Antibodies used	TAP Tag Antibody (CAB1001), Polyclonal, Rabbit, from Thermo Scientific<br>Secondary antibody : SAB1003, Goat, anti-rabbit HRP conjugate from Thermo Scientific
Validation	This primary antibody was used to detect an heterologous epitope. No signal is obtained whith WT strains, or uninduced in the case of inducible expression. See for instance Rastogi, S. K. et al. Ifu5, a WW domain-containing protein interacts with Efg1 to achieve coordination of normoxic and hypoxic functions to influence pathogenicity traits in Candida albicans. Cell Microbiol e13140 (2019) doi:10.1111/cmi.13140.

Animals and other organisms

Policy information about studies involving animals; ARRIVE guidelines recommended for reporting animal research

Laboratory animals	Female BALB/c mice at 6-8 weeks of age
Wild animals	No wild animals were used in the study
Field-collected samples	No field-collected samples were used in the study
Ethics oversight	All procedures were approved by a local ethics committee and the French Ministry of Higher Education, Research and Innovation (Authorization N°8384) in accordance with the European Communities Council Directive (86/609/EU) and the European Union guidelines.

Note that full information on the approval of the study protocol must also be provided in the manuscript.
